# Complete Genome Sequence of Cluster C2 *Bacillus* Phage Maceta

**DOI:** 10.1128/MRA.01298-18

**Published:** 2018-12-13

**Authors:** Elizabeth Greguske

**Affiliations:** a Department of Biology, Saint Anselm College, Manchester, New Hampshire, USA; Georgia Institute of Technology

## Abstract

*Bacillus* phage Maceta was isolated from the soil of commercially purchased annual flowers using the host Bacillus thuringiensis serovar kurstaki. Isolated DNA was then sequenced and annotated. Maceta has a relatively small genome, containing 45,023 bp, and shares an average nucleotide identity of 96% with other cluster C2 *Bacillus* phage.

## Abstract (precis)

*Bacillus* phage Maceta was isolated from the soil of commercially purchased annual flowers using the host Bacillus thuringiensis serovar kurstaki. Isolated DNA was then sequenced and annotated.

## ANNOUNCEMENT

Maceta is a novel *Bacillus* bacteriophage isolated, characterized, and annotated by an undergraduate student research lab associated with the Howard Hughes Medical Institute (HHMI) Phage Hunters (SEA-PHAGES) program (1). Maceta, named for the flower pot from which the soil was sampled, was isolated in 2014 using Bacillus thuringiensis serovar kurstaki as the host bacterium. Both the soil and the annual flowers in the outdoor pot (Saint Anselm College, Manchester, NH, USA) were commercially purchased and then planted by groundskeepers 2 weeks prior to sampling. The soil sample was added to 45 ml of Trypticase soy broth (TSB) and 5 ml of log-phase Bacillus thuringiensis serovar kurstaki, mixed well, and then grown overnight at 37°C. The supernatant was collected and centrifuged at 3,000 rpm for 10 min to pellet the remaining soil and Bacillus thuringiensis serovar kurstaki. The pellet was discarded, and the remaining liquid was sterile filtered through a 22-µm filter with vacuum suction (2). The resulting filtrate was tested for sufficient phage concentration (>10^8^ PFU/ml) via serial dilution and plate counts. DNA was isolated from purified phage using a Promega Wizard DNA kit and sequenced via the MiSeq Illumina platform at the Hubbard Center for Genome Studies (University of New Hampshire [UNH], Durham, NH, USA), resulting in 9,530,240 paired reads, with a read length average of 151 bp. Trimmomatic v0.33 with default settings was used to trim the reads (3). Geneious v10.2 with default settings (4) was used to assemble the genome, and the quality of the assembly was assessed by Quast v4.0 (5). The genome was then further refined using Geneious v10.2 with default settings except for sensitivity, which was set to highest sensitivity/slow. The average depth of coverage was 3,984.2×, with no areas of poor coverage noted. Maceta was autoannotated in Geneious v10.2 with default settings, using *Bacillus* phage Bastille, GenBank accession number JF966203 (6), for comparison. Maceta was then manually cross-checked against 4 other closely related cluster C *Bacillus* phage genomes, namely CAM003, Evoli, Vinny, and Anthony, registered under GenBank accession numbers KJ489397 (7), KJ489398 (7), KU737346 (8), and MF498901 (9), respectively.

Maceta has a relatively short genome compared with those of other *Bacillus* phage (10), containing 45,023 bp with a GC content of 40.0%. The genome contains 46 predicted genes, 37 of which are protein-coding regions and 9 of which are tRNA-coding regions. Of the 37 protein-coding regions, 19 were assigned a function, all of which are involved in capsid and tail production and assembly or host cell attachment and penetration. No endonucleases were identified, strongly suggesting that Maceta has an obligatory lytic life cycle and is unable to undergo the lysogenic life cycle. Maceta contains 9 tRNAs, 8 of which are clustered together near the end of the linear genome. The remaining tRNA is sandwiched between a tail fiber hydrolase protein-coding sequence and an HK97 family portal protein-coding sequence.

Cluster assignment was based on average nucleotide identity (ANI) as previously described (7, 10). Maceta had a whole-genome ANI of 96% with 3 C2 phage (6, 9), as determined by BLASTn (11), firmly placing Maceta in this widespread cluster.

### Data availability.

The complete genome sequence of *Bacillus* phage Maceta is available in GenBank under accession number MH538296. Raw sequence reads are available in SRA under BioProject accession number PRJNA498083. Maceta is also registered with the HHMI Phage Hunters Program at bacillus.phagesdb.org (12).
